# Progress of radiomics research on white matter hyperintensity lesions

**DOI:** 10.3389/fneur.2025.1647724

**Published:** 2025-08-26

**Authors:** Lin Du, Lieping Wang, Gang Shen, Min Zeng, Desheng Li, Weiguo Li

**Affiliations:** ^1^Department of Radiology, Chongqing Tongliang District Hospital of Traditional Chinese Medicine, Chongqing, China; ^2^Department of Acupuncture and Pain, Chongqing Tongliang District Hospital of Traditional Chinese Medicine, Chongqing, China

**Keywords:** white matter hyperintensity (WMH), cerebral small vessel disease (CSVD), radiomics, cognitive impairment, stroke risk

## Abstract

White matter hyperintensity (WMH) is the core imaging hallmark of cerebral small vessel disease (CSVD). This phenomenon is closely related to nervous system damage, such as cognitive impairment, dementia and increased risk of stroke. However, traditional diagnostic methods have significant limitations in terms of quantitative assessment, analysis of pathological mechanisms, and clinical decision support, which severely restrict their clinical application. Through high-throughput feature extraction and comprehensive analysis of clinical, laboratory, histological, and genomic data, radiomics in its current form can not only achieve the high-precision identification and staging of WMH but also help to reveal its pathological mechanism, which has shown important value in the diagnosis, prognosis, and evaluation of WMH-related diseases. Against this backdrop, we strictly adhered to the norms of systematic literature reviews, conducting a comprehensive and transparent literature search. We also thoroughly reviewed the data using a predefined strategy and strict inclusion/exclusion criteria (detailed in the text). This article systematically reviews the progress of radiomics research in characterizing the pathological mechanism of WMH and in the early identification, classification and prognostic evaluation of related diseases, aiming to provide a theoretical basis and a technical reference for the early identification of high-risk groups, the optimization of diagnosis and treatment decision-making, and the practice of collaborative patient management.

## 1 Introduction

White matter hyperintensity (WMH) is an imaging feature based on magnetic resonance imaging (MRI). WMH lesions present high signal intensity on T2-weighted imaging (T2WI) or fluid-attenuated inversion recovery (FLAIR) sequences and are mainly distributed in the bilateral deep brain and periventricular white matter, symmetrically or asymmetrically (1). Clinically, WMH is recognized as the core imaging marker of cerebral small vessel disease (CSVD) (1, 2), which is closely related to nervous system damage, such as cognitive dysfunction, dementia and increased risk of stroke (3–5). Furthermore, some researchers have reported that WMH in acute cerebrovascular diseases is mainly associated with the subtype of lacunar infarction (6). According to the literature, WMH increases the risk of cognitive impairment and all-cause dementia (ACD) by 14% while increasing the risk of Alzheimer’s disease (AD) by 25% and the risk of vascular dementia (VaD) by 73% (5). Other studies have shown that WMH can be used as an independent predictor of stroke (7), and its severity is significantly associated with an increased risk of ischemic and hemorrhagic stroke (7, 8). In clinical practice, early and accurate identification and staging of WMH are of great clinical importance for the diagnosis and prognosis of the disease.

MRI findings combined with Fazekas scale scores are commonly used to assess the severity of WMH (9–11). However, owing to the shortcomings of artificial subjectivity, quantitative bias and lack of pathological interpretation, their value in clinical and scientific research is limited. In recent years, radiomics has been used to extract quantitative and highly reproducible information from CT, MR, PET/SPECT and other imaging images; capture tissue and lesion characteristics; and comprehensively evaluate the obtained feature patterns by combining clinical, laboratory, histological, and genomic data. It has shown broad application prospects in analyzing the pathophysiology of diseases and predicting therapeutic effects, and it has also shown great potential in research on WMH-related diseases (12–14). This article reviews the progress of radiomics research on the pathological mechanism of WMH and the early identification, classification and prognostic evaluation of related diseases to provide a clinical basis for the formulation of early intervention strategies.

## 2 Methods

This study strictly adhered to the systematic literature search protocol of the Preferred Reporting Items for Systematic Review and Meta-Analyses (PRISMA) guidelines (15).

**Figure 1:**
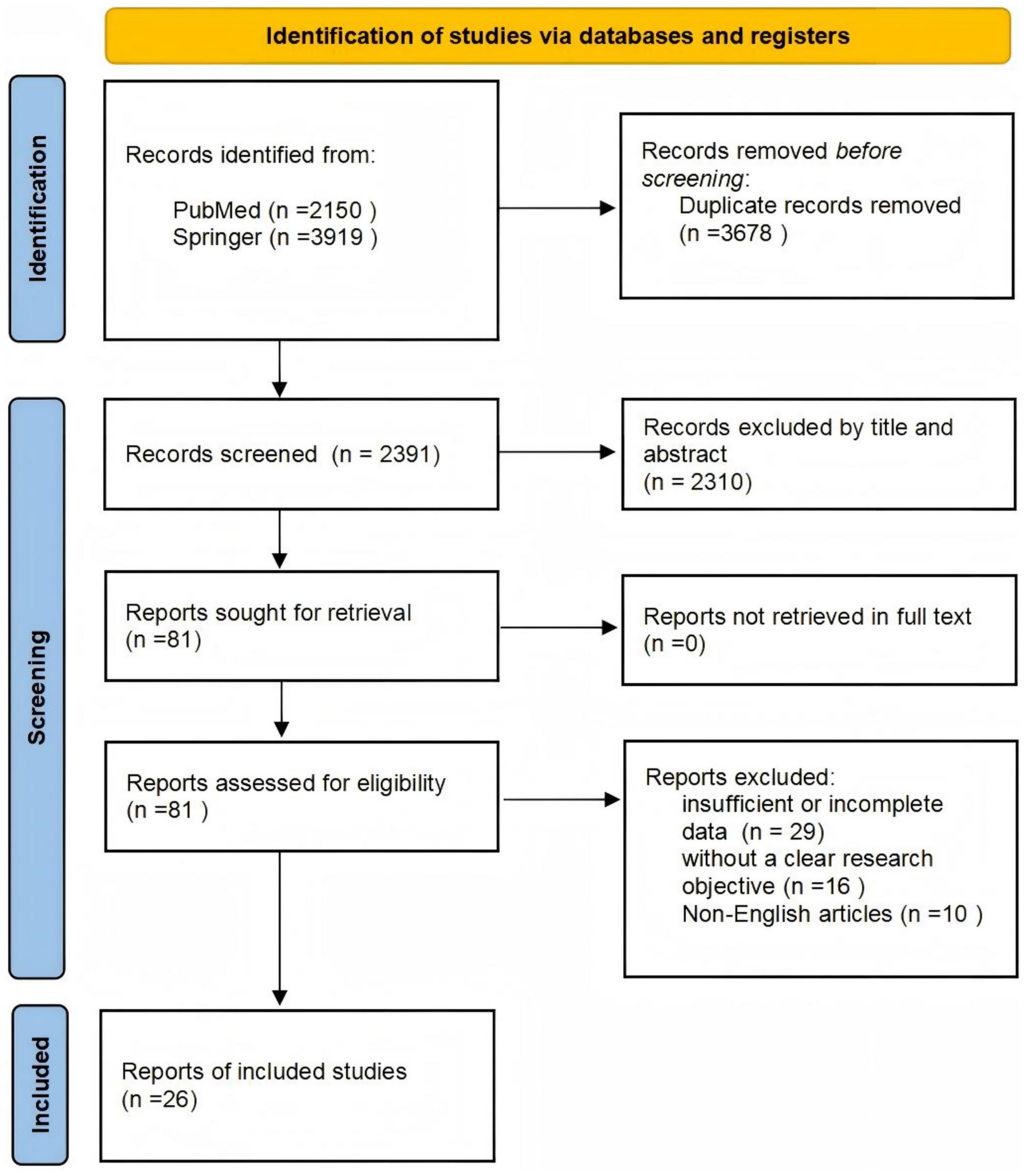
A schematic workflow of the study.

During the data collection stage, we conducted a detailed and comprehensive search of the PubMed and Springer databases using logical combinations of keywords to ensure the reliability and coverage of the data. The adopted search strategy included terms such as “white matter hyperintensity,” “cerebral small vessel disease,” “radiomics,” “stroke,” “cognitive dysfunction,” “dementia,” “Alzheimer’s disease” and “Parkinson’s disease.” The references obtained through manual search were used to identify more published articles. The research is limited to non-conference academic publications published in English. The date of this literature search ranged from January 2000 to March 2025. A total of 6,069 records were ultimately retrieved. After careful adjustment and optimization of the search strategy, 26 records were deemed relevant to the research topic and were included in the study. The following is a schematic workflow of the study (Figure 1). The neurology literature feature extraction is shown in Table 1.

**Table 1:** The neurological literature feature extraction table.

Number	Authors/Ref.	Paper title	Study design	Sample size (*n*)	Research objectives	Main conclusions
1	Etherton MR et al. (7)	Normal-Appearing White Matter Microstructural Injury Is Associated with White Matter Hyperintensity Burden in Acute Ischemic Stroke	A retrospective study	319 AIS patients (mean age 64.9 ± 15.9 years) with MRI (DTI/FLAIR) within 48 h of onset.	To characterize diffusion tensor imaging (DTI) features of normal-appearing white matter (NAWM) and white matter hyperintensity (WMH) in acute ischemic stroke (AIS) patients, and explore associations between NAWM microstructural injury and WMH burden.	Normal-appearing white matter axial diffusivity increases with age and is an independent predictor of white matter hyperintensity volume in acute ischemic stroke.
2	Giese AK et al. (53)	White Matter Hyperintensity Burden in Acute Stroke Patients Differs by Ischemic Stroke Subtype	A retrospective study	3,301 AIS patients, with 2,529 patients analyzed after quality control of FLAIR MRI data.	To examine etiologic stroke subtypes and vascular risk factor profiles and their association with white matter hyperintensity (WMH) burden in patients hospitalized for acute ischemic stroke (AIS).	Vascular risk factor profiles and extent of WMH burden differ by Causative Classification of Ischemic Stroke (CCS)subtype, with the highest lesion burden detected in patients with small artery occlusion (SAO). These findings further support the small vessel hypothesis of WMH lesions detected on brain MRI of patients with ischemic stroke.
3	Gupta R et al. (50)	Quality Assessment of Radiomics Studies on Functional Outcomes After Acute Ischemic Stroke-A Systematic Review	Review	14 Studies using radiomics-extracted features to predict functional outcomes among AIS patients using the modified Rankin Score (mRS) were included.	To assess the quality of existing studies which use radiomics methods to predict functional outcomes in patients following AIS.	Included studies showed low-to-moderate quality. As per the QUADAS-2, 6/14 (42.9%) studies had risk of bias concern and 0/14 (0%) had applicability concern.
4	Guo Y et al. (54)	Novel Survival Features Generated by Clinical Text Information and Radiomics Features May Improve the Prediction of Ischemic Stroke Outcome.	A retrospective study	A total of 80 DSC-PWI images from 56 patients with ischemic stroke were included.	To evaluate the performance of clinical text information (CTI), radiomics features, and survival features (SurvF) for predicting functional outcomes of patients with ischemic stroke.	The combination of mRSRF and CTI can accurately predict functional outcomes in ischemic stroke patients with proper machine learning models. Moreover, combining SurvF will improve the prediction effect compared with the original features.
5	Tang T-y et al. (55)	Penumbra-Based Radiomics Signature as Prognostic Biomarkers for Thrombolysis of Acute Ischemic Stroke Patients: A Multicenter Cohort Study.	A multicenter retrospective study	168 AIS patients within 9 h after onset were collected from seven hospitals and divided into a training dataset and an external validation dataset.	To develop a radiomics signature (R score) as prognostic biomarkers based on penumbra quantification and to validate the radiomics nomogram to predict the clinical outcomes for thrombolysis for acute ischemic stroke (AIS) patients.	The radiomics signature is an independent biomarker for estimating the clinical outcomes in AIS patients. By improving the individualized prediction of the clinical outcome for AIS patients 3 months after onset, the radiomics nomogram adds more value to the current clinical decision-making process.
6	Guo K et al. (56)	Machine Learning-Based Nomogram: Integrating Mri Radiomics and Clinical Indicators for Prognostic Assessment in Acute Ischemic Stroke.	A retrospective study	506 AIS patients involved	To develop and validate a nomogram that combines a multi-MRI radiomics signature with clinical factors for predicting the prognosis of AIS.	The study underscores the efficacy of the clinical-radiomics model in forecasting AIS prognosis, offering a significant leap toward more individualized and effective healthcare solutions.
7	Xia Y et al. (57)	Machine Learning Prediction Model for Functional Prognosis of Acute Ischemic Stroke Based on Mri Radiomics of White Matter Hyperintensities.	A retrospective study	202 inpatients with acute anterior circulation ischemic stroke from the Department of Neurology.	To explore the value of a nomogram that integrates clinical factors and MRI white matter hyperintensities (WMH) radiomics features in predicting the prognosis at 90 days for patients with acute ischemic stroke (AIS).	The FLAIR sequence-based WMH radiomics approach demonstrates effective prediction of the 90-day functional prognosis in patients with AIS. The integration of TWMH radiomics and clinical factors in a combined model exhibits superior performance.
8	Bonkhoff AK et al. (58)	Association of Stroke Lesion Pattern and White Matter Hyperintensity Burden with Stroke Severity and Outcome.	A retrospective study	928 AIS patients (severity analysis) and 698 followed-up patients (outcome analysis) from the MRI-GENIE cohort	To examine whether high white matter hyperintensity (WMH) burden is associated with greater stroke severity and worse functional outcomes in lesion pattern-specific ways.	1. High WMH burden exacerbated severity for lesions in left insular-inferior frontal (language) and right temporo-parietal (attention) regions 2. High WMH burden independently increased unfavorable outcomes. 3. Bilateral subcortical lesions (thalamus, internal capsule) were strongest predictors of severity/outcomes, independent of WMH.
9	Bretzner M, et al. (59)	MRI Radiomic Signature of White Matter Hyperintensities Is Associated with Clinical Phenotypes.	A retrospective study	A multi-center cohort of 4,163 acute ischemic stroke (AIS) patients (mean age 62.8 years, 42% female) with T2-FLAIR MRI.	To evaluate radiomics for predicting white matter hyperintensity (WMH) burden and assessing brain structural integrity using conventional MRI. To uncover associations between radiomic features of WMH and clinical phenotypes.	Radiomics extracted from T2-FLAIR images of AIS patients capture microstructural damage of the cerebral parenchyma and correlate with clinical phenotypes, suggesting different radiographical textural abnormalities per cardiovascular risk profile.
10	Meng F, et al. (16)	Research Progress on Mri for White Matter Hyperintensity of Presumed Vascular Origin and Cognitive Impairment.	Review	Middle-aged and elderly individuals	To review the association between WMH and cognitive impairment and the application of dynamic contrast-enhanced MRI, structural MRI, diffusion tensor imaging, 3D-arterial spin labeling, intravoxel incoherent motion, magnetic resonance spectroscopy, and resting-state functional MRI for examining WMH and cognitive impairment.	Multimodal MRI enables non-invasive assessment of structural, metabolic, and functional abnormalities in WMH, providing an important tool for early diagnosis, pathophysiological research, and treatment monitoring of cognitive impairment.
11	Pasi M, et al. (60)	Clinical Relevance of Cerebral Small Vessel Diseases. Stroke	Review	Middle-aged and elderly populations	To review the main clinical phenotypes of cerebral small vessel disease (SVD), including acute ischemic/hemorrhagic events and cognitive impairment, explore the clinical significance of MRI markers (e.g., white matter hyperintensities, lacunes, microbleeds), and analyze the impact of asymptomatic SVD on disabling conditions such as stroke and dementia.	Associated with elevated ischemic/hemorrhagic stroke risk; severe WMH triples dementia risk.
12	Grau-Olivares M, et al. (61)	Mild Cognitive Impairment in Stroke Patients with Ischemic Cerebral Small-Vessel Disease: A Forerunner of Vascular Dementia?	Review	Middle-aged and elderly patients with ischemic stroke Asymptomatic elderly individuals with MRI-detected SVD markers and patients with mild cognitive impairment (MCI) or early dementia.	To investigate the association between ischemic cerebral small vessel disease (SVD) and cognitive impairment, and to evaluate whether vascular mild cognitive impairment (vMCI) is a precursor of subcortical vascular dementia.	Severe WMH (particularly periventricular) independently predicts slowed processing speed and memory decline, strongly associated with dementia risk.
13	Tang L, et al. (65)	Individualized Prediction of Early Alzheimer’s Disease Based on Magnetic Resonance Imaging Radiomics, Clinical, and Laboratory Examinations: A 60-Month Follow-up Study.	Retrospective cohort study	162 mild cognitive impairment (MCI) patients	To develop and validate radiomics models and multipredictor nomogram for predicting the time to progression (TTP) from MCI to AD	The prediction of individual TTP from MCI to AD could be accurately conducted using the radiomics clinical-laboratory model and multipredictor nomogram.
14	Dadar M, et al. (66)	White Matter Hyperintensity Distribution Differences in Aging and Neurodegenerative Disease Cohorts.	Cross-sectional observational study	976 participants from the COMPASS-ND cohort of the Canadian Consortium on Neurodegeneration in Aging (CCNA).	To compare the distribution characteristics of white matter hyperintensities (WMH) in aging and neurodegenerative disease cohorts, including prevalence, regional differences, sex-specific patterns, and hemispheric asymmetry, and explore associations with cognitive impairment.	There were distinct differences in WMH prevalence and distribution across diagnostic groups, sexes, and in terms of asymmetry. WMH burden was significantly greater in all neurodegenerative dementia groups, likely encompassing areas exclusively impacted by neurodegeneration as well as areas related to cerebrovascular disease pathology.
15	Garnier-Crussard A, et al. (67)	White Matter Hyperintensity Topography in Alzheimer’s Disease and Links to Cognition.	Cross-sectional observational study	54 cognitively impaired amyloid beta– positive AD (Aβpos-AD), compared to 40 cognitively unimpaired amyloid beta– negative older controls (Aβneg-controls) matched for vascular risk factors.	To investigate the topographic distribution of white matter hyperintensities (WMH) in Alzheimer’s disease (AD) and their independent associations with cognitive function, excluding the effects of amyloid deposition and brain atrophy.	1. AD patients had larger WMH volumes than controls in all regions, with the most significant increase in the splenium of the corpus callosum (S-CC). 2. Total WMH volume and S-CC WMH were strongly associated with cognitive decline (e.g., executive function, memory) in AD, independent of Aβ deposition and atrophy.
16	Fiford CM, et al. (68)	Automated White Matter Hyperintensity Segmentation Using Bayesian Model Selection: Assessment and Correlations with Cognitive Change.	Retrospective cohort study	Magnetic resonance images from 30 control and 30 AD participants	To evaluate the performance of Bayesian Model Selection (BaMoS) for automated white matter hyperintensity (WMH) segmentation and validate its ability to predict longitudinal cognitive decline in individuals with normal cognition, mild cognitive impairment (MCI), and Alzheimer’s disease (AD).	BaMoS is suitable for large-scale multi-center studies, providing reliable quantification of WMH to explore its role in cognitive impairment.
17	Carvalho de Abreu DC, et al. (3)	Is White Matter Hyperintensity Burden Associated with Cognitive and Motor Impairment in Patients with Parkinson’s Disease? A Systematic Review and Meta-Analysis.	A systematic review and meta-analysis	Fifty eligible studies were included, involving PD patients, PD-MCI patients, PDD patients, and healthy controls.	To examine the association between WMH and cognitive and motor performance in PD through a systematic review and meta-analysis.	WMH burden appears to increase with PD worse cognitive and motor status in PD
18	Hou M, et al. (70)	Characteristics of Cognitive Impairment and Their Relationship with Total Cerebral Small Vascular Disease Score in Parkinson’s Disease.	A retrospective study	174 idiopathic PD patients who underwent brain magnetic resonance imaging (MRI) were recruited	To investigate the characteristics of cognitive dysfunctions and their relationship with total cerebral small vascular disease (CSVD) in Parkinson’s disease (PD)	CSVD can independently contribute to cognitive decline in PD and cause damage in specific cognitive domains. Promoting neurovascular health may help preserve cognitive functions in PD.
19	Huang X, et al. (76)	Periventricular White Matter Hyperintensity Burden and Cognitive Impairment in Early Parkinson’s Disease.	Cross-sectional study (baseline data analysis of a prospective cohort)	175 non-demented early PD patients who had undergone baseline brain MRI were included.	Quantified the total brain and periventricular white matter hyperintensities (WMHs) burdens in patients with early Parkinson disease (PD) and explored their associations with cardiovascular risk factors and cognitive performance.	Periventricular WMHs burden was independently associated with PD-MCI, as well as worse executive function and visuospatial function.
20	Wu H, et al. (77)	Regional White Matter Hyperintensity Volume in Parkinson’s Disease and Associations with the Motor Signs.	Combined cross-sectional and longitudinal study	A total of 50 PD participants and 47 age- and gender-matched controls were enrolled.	To investigate the association between regional white matter hyperintensity (WMH) volumes and Parkinson’s disease (PD), and to assess their impact on motor signs through cross-sectional and longitudinal analyses.	PD participants in this study were characterized by greater WMH at the occipital region, and greater occipital WMH volume had cross-sectional associations with worse motor signs, while its longitudinal impact on motor signs progression was limited
21	Liu H, et al. (78)	The Influence of White Matter Hyperintensity on Cognitive Impairment in Parkinson’s Disease.	Meta-analysis	15 eligible studies	To review systematically and to identify the relationship between the severity and location of white matter hyperintensities (WMHs) and the degree of cognitive decline in patients with Parkinson’s disease (PD).	WMHs might be imaging markers for cognitive impairment in PDD but not in PD-MCI, regardless of age, vascular risk factors, or race. Further prospective studies are needed to validate the conclusions.
22	Shu Z, et al. (48)	An Integrative Nomogram for Identifying Early-Stage Parkinson’s Disease Using Non-Motor Symptoms and White Matter-Based Radiomics Biomarkers from Whole-Brain Mri.	Retrospective cohort study	336 participants (168 PD patients, 168 healthy controls)	To develop and validate an integrative nomogram based on white matter (WM) radiomics biomarkers and nonmotor symptoms for the identification of early-stage Parkinson’s disease (PD).	This integrative nomogram is a new potential method to identify patients with early-stage PD.
23	Rektor I, et al. (79)	White Matter Alterations in Parkinson’s Disease with Normal Cognition Precede Gray Matter Atrophy.	Cross-sectional case–control study	Twenty PD patients and twenty-one healthy controls (HC)	To detect gray matter (GM) and white matter (WM) changes in PD patients without cognitive impairment.	WM microstructural damage occurs early in PD prior to overt GM atrophy, suggesting WM alterations as a sensitive biomarker for early PD.
24	Shu ZY, et al. (80)	Predicting the Progression of Parkinson’s Disease Using Conventional Mri and Machine Learning: An Application of Radiomic Biomarkers in Whole-Brain White Matter.	Retrospective cohort study	144 PD patients (72 progressive, 72 stable)	To develop and validate a radiomics model based on whole-brain white matter and clinical features to predict the progression of Parkinson disease (PD).	Conventional structural MRI can predict the progression of PD. This work also supports the use of a simple radiomics signature built from whole-brain white matter features as a useful tool for the assessment and monitoring of PD progression.
25	Haliasos N, et al. (81)	Personalizing Deep Brain Stimulation Therapy for Parkinson’s Disease with Whole-Brain Mri Radiomics and Machine Learning.	Retrospective cohort study	A total of 120 PD patients underwent Deep brain stimulation (DBS) of the subthalamic nucleus. T	To develop a machine learning-driven predictive model for DBS patient selection using whole-brain white matter radiomics and common clinical variables.	Machine learning models can be used in clinical decision support tools which can deliver true personalized therapy recommendations for PD patients.
26	Tubi MA, et al. (81)	White Matter Hyperintensities and Their Relationship to Cognition: Effects of Segmentation Algorithm.	Cross-sectional study	260 non-demented participants	To investigate how different WMH segmentation algorithms affect the relationship between WMH volume and cognitive function, and to analyze whether Alzheimer’s disease (AD)-specific pathology masks the cognitive effects of WMH.	AD neuropathology may mask WMH effects on clinical diagnosis and cognition.

The inclusion criteria for the literature were online publications specifically related to the abovementioned topic and those published in English. The exclusion criteria were duplicate publications, studies with insufficient or incomplete data for which relevant information could not be extracted, studies without a clear research objective, and studies published in languages other than English. The researchers used EndNote X9 reference management software to eliminate duplicate studies. Two researchers independently screened the titles and abstracts to determine whether they met the inclusion criteria. Disagreements were resolved by discussion; if there was still disagreement, a third researcher was consulted.

## 3 Manuscript formatting

### 3.1 Overview of white matter hyperintensity in the brain

#### 3.1.1 Epidemiology

The prevalence of WMH increases with age and ranges from approximately 20–100% (16–18). Wide variation exists due to differences in the race, sex, and age of the subjects and the different research methods used (such as examination methods, observation sites, and evaluation criteria). The prevalence of WMH varies significantly among different races, with studies showing higher prevalence and progression rates of WMH among black people than white people (19, 20). Another study reported that the prevalence of confluent WMH in the Han Chinese population was significantly greater than that in Australian Caucasians (21). Differences also exist in the incidence of WMH in patients according to sex. Lohner et al. (22) reported that the rate of progression and the proportion of severe WMH lesions (Fazekas ≥ 2) in postmenopausal women were significantly greater than those in contemporary men, and women with uncontrolled hypertension were found to have a greater WMH burden than men, which was unrelated to menopausal status. In middle-aged people, the prevalence of WMH is approximately 20–50% (17). Among people aged 60–70 years, 87% had deep white matter hyperintensity (DWMH), and 68% had periventricular white matter hyperintensity (PVWMH). The prevalence of DWMH and PVWMH reached 100 and 95%, respectively, in people aged 80–90 years (16, 18).

#### 3.1.2 Risk factors

The development of WMH is related to the interaction of multiple systems, and the main risk factors include vascular endothelial dysfunction and hemodynamic disorders caused by hypertension, diabetes, atherosclerosis and smoking (17, 23–26); cerebral metabolic abnormalities and vasomotor disorders caused by hyperlipidemia, migraine, and sleep disorders (27–29); and genetic diseases (such as adrenoleukodystrophy) and immune-mediated inflammatory diseases (such as multiple sclerosis) (30–32).

#### 3.1.3 Pathological mechanism

The pathogenesis of WMH is complex, and the core pathological changes are characterized by demyelination, oligodendrocyte apoptosis, axonal injury and reactive gliosis (16). The current mainstream hypotheses include the following four categories: (1) Hypoperfusion and ischemic injury: Factors such as arterial stenosis and endothelial dysfunction lead to chronic white matter hypoperfusion, which leads to disordered oligodendrocyte energy metabolism and ischemic demyelination (16, 23, 33). (2) Blood–brain barrier (BBB) disruption: Vascular permeability is abnormally increased, and plasma components (such as inflammatory factors and fibrinogen) leak into the brain parenchyma, directly damaging myelin structure and reducing white matter fiber density (34). (3) Vein collagen hyperplasia and microcirculation disorder: Venous ischemia, collagen deposition in venules around the ventricles and obstruction of flow in the jugular vein occur, resulting in intensified microcirculation blockage and obstacles to the removal of metabolic waste (35). (4) Hypoperfusion, BBB destruction and other pathological processes activate microglia and release proinflammatory factors, which further accelerate white matter damage (32).

### 3.2 Diagnostic methods and grading criteria for WMH

#### 3.2.1 Diagnostic MRI techniques for WMH

MRI is a noninvasive, radiation-free and high-resolution method to evaluate the brain structure and function of patients with WMH. In clinical practice, the MRI techniques related to the diagnosis of WMH mainly include conventional plain MRI (T1-weighted imaging (T1WI), T2WI and T2-FLAIR) (1), arterial spin labeling (ASL) (36), diffusion tensor imaging (DTI) (37–39) and diffusion kurtosis imaging (DKI) (39–42).

T2WI and T2-FLAIR are highly sensitive in detecting WMH. In particular, 3D-T2-FLAIR, which was developed on the basis of T2-FLAIR, has characteristics including high resolution and fast imaging speed, which are highly valuable for the detection of WMH, but this modality cannot define the pathogenesis of the lesions and the pathological changes associated with them. ASL can directly and quantitatively measure cerebral blood flow (CBF) by labeling water molecules in arterial blood as an endogenous tracer. It has high value for evaluating WMH induced by hypoperfusion, but it is less sensitive to small lesions and is easily disturbed by motion artifacts. DTI can quantify the microstructure and integrity of white matter by detecting the diffusion characteristics of water molecules in white matter fiber tracts, which has the advantages of noninvasiveness, safety, high sensitivity, three-dimensional visualization, fiber tracking and dynamic monitoring. However, there is confusion and interference in the crossing area of nerve fiber tracts, which affects the measurement results of WMH lesions. DKI introduces a fourth-order tensor model, which can more accurately analyze the non-Gaussian characteristics of water molecular diffusion and compensate for the shortcomings of DTI, which is based on the Gaussian diffusion model only. Compared with DTI, DKI is more sensitive to tissue microstructural changes, and the measurement results in the crossing area of nerve fibers are more reliable, which improves the ability to detect early lesions. However, owing to the limited image resolution, the display of WMH lesions is greatly limited. Moreover, DTI and DKI require considerable equipment, and the interpretation of parameters is complex, which makes these techniques less popular in clinical practice.

#### 3.2.2 Grading criteria

**Figure 2:**
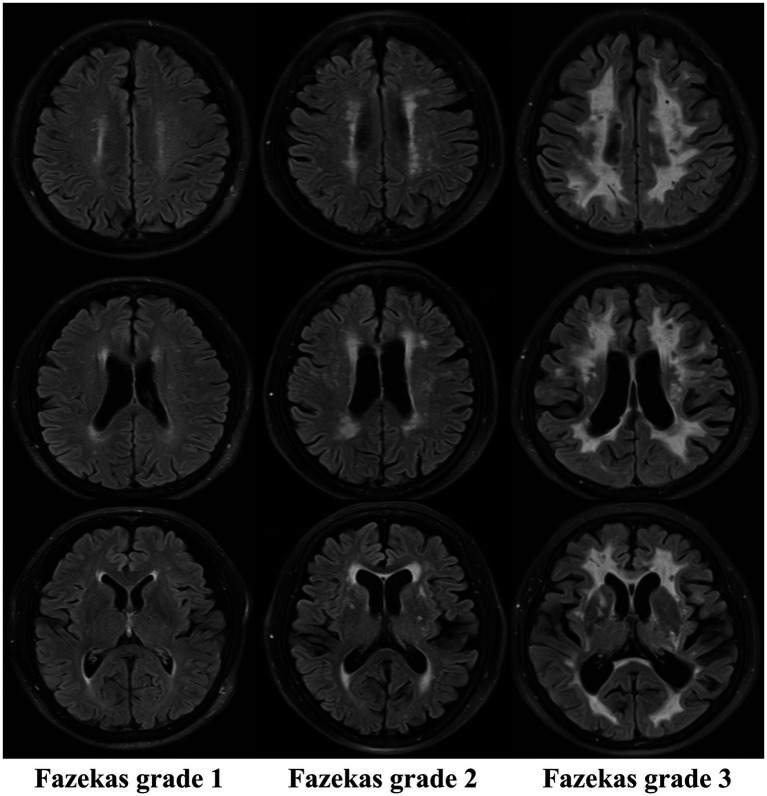
Characteristic MR imaging findings on white matter hyperintensity lesions (Fazekas).

The Fazekas grading system is the classical imaging standard for assessing the severity of WMH (9–11, 43). The most common classification of WMHs is location, as periventricular white matter hyperintensities (PWMHs) around the lateral ventricles and deep white matter hyperintensities (DWMHs) in the deep part of the subcortical white matter are assigned grades 0–3. According to the morphology of the lesions, PWMHs are divided into no lesions (0 grade), cap or pencil-thin lesions (1 grade), smooth halo lesions (2 grade) and irregular extension into the deep white matter (3 grade). DWMH scores are assigned as follows: no lesion (0 grade), punctate lesions (1 grade), lesions beginning to fuse into plaque (2 grade) and large areas of lesions fused into patches (3 grade). The modified Fazekas classification was as follows: grade 1 (scattered punctate lesions with a speckle-like appearance), grade 2 (some lesions fused to a plaque), and grade 3 (large lesions fused to a patch). The characteristic MR imaging findings on white matter hyperintensity lesions refer to Figure 2 for details.

The Fazekas classification is easy to apply and is the most widely used method in clinical practice, but it is prone to subjective effects arising from equipment and manual interpretation. Therefore, we need to introduce a more objective, sensitive, accurate and efficient tool to reduce human error, improve the display rate of lesions, accurately localize and quantify lesions, reveal the etiology and pathological changes of the disease, and lay a solid foundation for clinical diagnosis and treatment.

### 3.3 Overview of radiomics

Radiomics is a technique for high-throughput extraction of quantitative features from CT, MR, PET/SPECT and other imaging modalities, combining clinical, genetic or molecular data and using machine learning or deep learning to construct predictive models. The process mainly includes the following five stages (12–14): (1) Data acquisition and preprocessing: Appropriate imaging modalities, such as CT, MRI, and PET, are selected, and the standardization of the data is ensured; (2) Region of interest (ROI) segmentation: There are three methods: manual, semiautomatic and automatic segmentation; (3) Feature extraction: high-throughput extraction of many valuable quantitative features from the segmented region of interest (ROI), including first-order statistical features, texture features, shape features and high-order features; (4) Feature selection and dimension reduction: To avoid model overfitting, it is necessary to screen out the features that are stable and related to the prediction target from the extracted features; and (5) Model construction and validation: According to the research purpose and data type, appropriate machine learning or deep learning algorithms were selected to construct prediction models (including logistic regression, support vector machines, random forests, neural networks, etc.) based on the selected features. Finally, cross-validation and external validation methods were used to evaluate the reliability and generalizability of the model. Radiomics can identify the microscopic characteristics of lesions by deep mining of the collected data, which provides a theoretical basis for the diagnosis, pathological analysis and prognostic evaluation of diseases.

### 3.4 The application and research progress of radiomics in WMH

#### 3.4.1 The application and progress of radiomics in basic pathological research on WMH

A longitudinal study of 51 general elderly individuals (44) demonstrated that radiomics texture analysis based on conventional MRI can be used for the early detection of white matter hyperintensity (WHM), providing patients with early indications of disease and more time for treatment. Shao et al. (45) reported that a radiomics model based on the WMH penumbra (WMHP) was significantly better than a whole-brain white matter (WBWM) model at predicting the progression and speed of WMH. The progression rate of WMH was correlated with only the rad-score from the WMHP ROI. The reason for this difference may be related to the evolution mechanism of WMH, which affects mainly the foci and gradually spreads to the periphery. The WMHP region has greater microstructural heterogeneity and is more sensitive to early dynamic changes. Hou et al. (46) constructed a radiomic hybrid model based on CT-fractional flow reserve (CT-FFR) and the pericoronary fat attenuation index (pFAI) to predict the progression of WMH. The pFAI↑ and CT-FFR↓ (the ↑ indicates “increase” and the ↓ indicates “decrease”) can predict white matter damage, and the common pathological mechanism of cardiac and cerebral microcirculation diseases is related to the inflammatory response. Shu et al. (47) developed a radiomics nomogram to screen out gray-level cooccurrence matrix (GLCM), form factor, and run-length matrix (RLM) features, which can noninvasively predict WMH progression in elderly patients aged ≥60 years. Shu et al. (48) constructed an integrated nomogram model of radiomics based on white matter, which could effectively distinguish scans of patients with Parkinson’s disease (PD) from scans without evidence of dopaminergic deficit (SWEDD). These findings suggest that white matter microstructural heterogeneity may underlie the pathological heterogeneity of PD and is associated with cognitive impairment. In addition, damage to white matter axons and the myelin sheath in PD patients occurs earlier than typical pathological changes, such as the loss of substantia nigra dopaminergic neurons. Ramon et al. (49) analyzed the textural features of different brain tissues and multisequence MR images through two traditional machine learning methods, and the results revealed that lacunar stroke was related to blood–brain barrier damage and that the white matter microstructural changes caused by it could be sensitively captured through textural features.

#### 3.4.2 The clinical application of radiomics in the study of WMH-related diseases

WMH can be observed in a variety of clinical diseases, including cerebrovascular diseases, demyelinating diseases, inflammatory reactions, tumors or other space-occupying lesions, brain trauma, and metabolic diseases. Among them, WMHs are particularly closely related to cerebrovascular diseases and neurodegenerative diseases.

##### 3.4.2.1 Application of radiomics in cerebrovascular diseases

Cerebrovascular accidents (CVAs) are the second leading cause of death and neurological disease, with the highest burden of death and disability in the world (50, 51). According to the pathological mechanism, CVAs can be divided into ischemic stroke and hemorrhagic stroke, and the former is the most common, accounting for 62.4% of cases (52). Studies have shown that ischemic stroke is associated with WMH (7, 53). At present, many studies have used radiomics technology to predict the individualized functional outcomes of patients with acute ischemic stroke (AIS) after discharge (50, 54–56), but relatively few studies have focused on the imaging features of WMH. Xia et al. (57) integrated radiomics and clinical factors to construct a combined model to predict the prognosis of acute ischemic stroke (AIS). The results showed that the radiomics model of total white matter hyperintensity (TWMH) using the support vector machine (SVM) classifier performed best in predicting the prognosis of AIS patients. Bonkhoff et al. (58) explored the associations between WMH burden and the severity and prognosis of stroke. The results revealed that a high WMH burden in specific lesion locations (such as the language-related area in the left hemisphere and the attention-related area in the right hemisphere) significantly aggravated the severity of the acute phase of stroke and increased the risk of poor long-term prognosis. Bretzner et al. (59) constructed a model for predicting WMH burden on the basis of conventional T2-FLAIR images of AIS patients and explored its association with clinical phenotypes. The results indicated that radiomics analysis of conventional T2-FLAIR images could capture microstructure damage in WMH and was significantly associated with clinical phenotypes such as age and cardiovascular risk factors.

Cerebral small vessel disease (CSVD) is a group of chronic and progressive diseases caused by a variety of factors that affect cerebral tiny arteries, capillaries, and venules. CSVD manifests as microstructure damage to white matter in the brain and has become the most common cause of vascular cognitive impairment, accounting for 45% of dementia cases (2, 38, 60). In clinical studies, subcortical vascular dementia is the most common type of dementia in patients with CSVD, accounting for 36–67% of all cases of vascular dementia (61). The signs of cerebral small vessel disease on conventional MRI include white matter hyperintensities, recent subcortical lacunar infarcts (clinically symptomatic), lacunes (clinically silent), cerebral microbleeds, prominent perivascular spaces, and cerebral atrophy (62). On MR images, WMH is a typical marker of CSVD, and its volume burden (WMH burden) can quantitatively reflect the cumulative degree of pathological damage, such as chronic cerebral ischemia, blood–brain barrier dysfunction and axonal demyelination (16, 60). Evidence (61) is available suggesting that the cognitive features of WMH include executive dysfunction, slowed information processing speed, and reduced language fluency. Moreover, the degree of cognitive impairment is significantly correlated with the location and quantity of white matter hyperintensity lesions in the brain. When patients progress to subcortical vascular dementia, the typical cognitive syndrome is executive dysfunction syndrome, which includes slowed information processing, memory deficits, behavioral and psychiatric symptoms, and language function that usually remains until the late stage of the disease. Owing to the diverse imaging manifestations and clinical symptoms of CSVD, radiomics research on WMH alone cannot be used as an independent diagnostic tool but can be used only as an auxiliary means. Therefore, further research in this field is needed.

##### 3.4.2.2 The application of radiomics in neurodegenerative diseases such as Alzheimer’s disease and Parkinson’s disease

Alzheimer’s disease (AD), a complex neurodegenerative disease, is not only the leading cause of dementia worldwide but also is associated with increasing incidence, high mortality, high medical expenditures and long-term care needs. It is rapidly becoming one of the most expensive, deadly and burdensome diseases of the century (63). Currently, studies on Alzheimer’s disease (AD) based on radiomics mainly focus on gray matter regions (64, 65). In recent years, an increasing number of studies have reported that the cognitive function impairment of AD patients is closely related to the volume of WMH (66, 67) and the distribution of brain regions (66, 67). However, Tang et al. (65) believed that the severity of white matter lesions is related to cognitive decline, but the volume of WMH is not an independent risk factor for the progression of MCI to AD. Fiford et al. (68) evaluated the performance of the Bayesian model selection (BaMoS) algorithm in the segmentation of WMH and analyzed its correlation with cognitive changes. The study revealed that the volume of WMH was significantly associated with subjective/significant memory concern (SMC), early mild cognitive impairment (EMCI), and late mild cognitive impairment (LMCI) in terms of cognitive decline but was not significantly associated with patients in the AD stage.

Based on the above research, consensus is still lacking on whether a correlation exists between the volume of WMH and cognitive impairment in AD patients, and some of the results are even contradictory. However, most scholars agree that the volume of WMH is related to cognitive impairment in AD patients. Radiomics is expected to make certain contributions to the study of the relationship between WMH in the brains of AD patients and changes in cognitive function.

Parkinson’s disease (PD) is a neurological disorder characterized by the progressive deterioration of motor and cognitive function with clinical manifestations of motor dysfunction and nonmotor symptoms, which seriously affects quality of life, and disease progression is associated with increased mortality (69, 70). In recent years, the application of radiomics in the research of Parkinson’s disease (PD) has made some progress, but most studies have focused on specific brain regions (such as the substantia nigra) or relied on multimodal combined applications (such as neuromelanin imaging, DTI, DKI, etc.), with some difficulty in clinical popularization and promotion (71–75). Numerous studies have shown that white matter hyperintensity (WMH), an imaging marker of white matter damage, may exacerbate cognitive and motor dysfunction (3, 76–78). The total WMH burden of PD patients is significantly greater than that of healthy controls, and its severity is positively correlated with the degree of cognitive decline (normal cognition → mild cognitive impairment [PD-MCI] → dementia (PDD)). A high WMH burden can also increase the risk of PD and related dementia (PDD) (3, 70, 76–78). Other studies have confirmed that in PD patients, white matter undergoes extensive structural changes before gray matter atrophy and cognitive impairment, and white matter (WM) changes may be a sensitive indicator of early PD (79). In clinical work, understanding the changes in WM microstructure and assessing the severity of PD are very important for the treatment and prognosis of PD patients. Shu et al. (80) constructed a joint model based on whole-brain white matter radiomics and clinical features to predict the progression of Parkinson’s disease (PD). Research has shown that the combined model can effectively predict the progression of PD, and a radiomic marker (rad-score) can also distinguish the severity of the disease. This method is low-cost, simple to perform, and can effectively predict the progression of PD, which is more suitable for clinical promotion. Shu et al. (48) developed a comprehensive nomogram model based on biomarkers and nonmotor symptoms of whole-brain MRI white matter radiomics for the identification of early Parkinson’s disease (PD). The results show that the model can effectively distinguish between typical PD patients and atypical PD patients (SWEDD: no evidence of dopaminergic deficiency on imaging) and provides a new method for the early diagnosis of PD. Haliasos et al. (81) developed a machine learning model based on radiomics of whole-brain white matter and clinical variables to predict the efficacy of deep brain stimulation (DBS) in patients with Parkinson’s disease. The results show that the combined model of machine learning can accurately screen patients who will benefit from DBS, which can be used as a clinical decision support tool to provide truly personalized treatment recommendations for PD patients. In summary, compared with other PD prediction models that rely on specific brain regions or complex MRI sequences, the radiomics model based on whole-brain white matter is more comprehensive and more likely to capture global brain structural changes, which compensates for the limited value of conventional MRI in the early diagnosis of PD, reduces the technical threshold, and facilitates adoption in primary medical care. However, there are still challenges, such as relatively small sample sizes and a lack of external validation, and the pathological mechanism of white matter features is not fully understood, which needs to be further explored and determined in future research.

### 3.5 Limitations

This article reviews the progress of radiomics research on the pathological mechanism of WMH and the early identification, classification and prognostic evaluation of related diseases. It provides a theoretical basis and technical reference for the early identification of high-risk populations, the optimization of diagnosis and treatment decisions, and collaborative patient management. Importantly, our research methods have two main limitations. First, we relied solely on the PubMed, and Springer databases for the comprehensive review of the literature, which may have led to confounding biases. Second, the correlation between the volume of WMH and cognitive impairment in AD patients remains inconclusive. According to previous scholars, this is related to the differences in the definition of lesion boundaries caused by different segmentation methods of WMH (82). Therefore, merely exploring the correlation between WMH and cognitive impairment in AD patients through radiomics may have certain limitations. Combining deep learning to optimize the segmentation method of WMH is expected to solve this problem, but this aspect was not covered in this article.

## 4 Summary and outlook

As the core imaging marker of cerebral small vessel disease, WMH is closely related to nervous system damage, such as cognitive impairment, dementia and increased risk of stroke. Early identification of WMH is highly important for the diagnosis, treatment and prognosis of related diseases. In recent years, radiomics has emerged as an innovative tool that effectively compensates for the shortcomings of traditional imaging diagnostic methods, providing new approaches for the early identification and pathological analysis of WMH. However, cognitive dysfunction is a common manifestation of a series of neurodegenerative diseases, and its correlation with WMH in the brain is still not fully understood. In the workflow of radiomics, accurate segmentation and precise quantification of WMH and surrounding brain regions are vital for determining the severity of disease. This requires the combination of multiple different deep learning algorithms to explore the optimal solution for WMH, which is conducive to the establishment of more accurate artificial intelligence diagnostic and prognostic models. Second, cognitive impairment is a dynamic process that is constantly changing. In the future, through dynamic omics, subgroup analysis, combined with metabolomics, a multicenter prospective experiment can be established for longitudinal analysis to construct a predictive model with broad interpretability to enhance the clinical application value of WMH in multiple fields, such as patient stratification, clinical decision making, and rehabilitation assistance.

## References

[1] Duering M Biessels GJ Brodtmann A Chen C Cordonnier C de Leeuw F-E. Neuroimaging standards for research into small vessel disease—advances since 2013. Lancet Neurol. (2023) 22:602–18. doi: 10.1016/s1474-4422(23)00131-x, PMID: 37236211

[2] An L Yuan W Wang Y Li S Zong C Gao Y. The positional relationship between lacunae and white matter Hyperintensity in patients with cerebral small vessel disease. Curr Neurovasc Res. (2023) 20:399–409. doi: 10.2174/1567202620666230721124707, PMID: 37488758

[3] Carvalho de Abreu DC Pieruccini-Faria F Son S Montero-Odasso M Camicioli R. Is white matter Hyperintensity burden associated with cognitive and motor impairment in patients with Parkinson’s disease? A systematic review and Meta-analysis. Neurosci Biobehav Rev. (2024) 161:105677. doi: 10.1016/j.neubiorev.2024.105677, PMID: 38636832

[4] Keller JA Sigurdsson S Klaassen K Hirschler L van Buchem MA Launer LJ. White matter Hyperintensity shape is associated with long-term dementia risk. Alzheimers Dement. (2023) 19:5632–41. doi: 10.1002/alz.13345, PMID: 37303267 PMC10713858

[5] Hu H-Y Ou Y-N Shen X-N Qu Y Ma Y-H Wang Z-T. White matter Hyperintensities and risks of cognitive impairment and dementia: a systematic review and Meta-analysis of 36 prospective studies. Neurosci Biobehav Rev. (2021) 120:16–27. doi: 10.1016/j.neubiorev.2020.11.007, PMID: 33188821

[6] Arboix A Massons J García-Eroles L Targa C Comes E Parra O. Nineteen-year trends in risk factors, clinical characteristics and prognosis in lacunar infarcts. Neuroepidemiology. (2010) 35:231–6. doi: 10.1159/000319460, PMID: 20861654

[7] Etherton MR Wu O Giese A-K Rost NS. Normal-appearing white matter microstructural injury is associated with white matter hyperintensity burden in acute ischemic stroke. Int J Stroke. (2019) 16:184–91. doi: 10.1177/1747493019895707, PMID: 31847795

[8] Park J-H Kwon SU Kwon HS Heo SH. Prior intracerebral hemorrhage and white matter Hyperintensity burden on recurrent stroke risk. Sci Rep. (2021) 11:3. doi: 10.1038/s41598-021-96809-3, PMID: 34465828 PMC8408204

[9] Pradeep A Raghavan S Przybelski SA Preboske GM Schwarz CG Lowe VJ. Can white matter Hyperintensities based Fazekas visual assessment scales inform about Alzheimer’s disease pathology in the population? Alzheimer’s Res Ther. (2024) 16:157. doi: 10.1186/s13195-024-01525-5, PMID: 38987827 PMC11234605

[10] Cedres N Ferreira D Machado A Shams S Sacuiu S Waern M. Predicting Fazekas scores from automatic segmentations of white matter signal abnormalities. Aging. (2020) 12:894–901. doi: 10.18632/aging.102662, PMID: 31927535 PMC6977667

[11] Andere A Jindal G Molino J Collins S Merck D Burton T. Volumetric white matter Hyperintensity ranges correspond to Fazekas scores on brain Mri. J Stroke Cerebrovasc Dis. (2022) 31:106333. doi: 10.1016/j.jstrokecerebrovasdis.2022.106333, PMID: 35158149

[12] Mayerhoefer ME Materka A Langs G Häggström I Szczypiński P Gibbs P. Introduction to Radiomics. J Nucl Med. (2020) 61:488–95. doi: 10.2967/jnumed.118.222893, PMID: 32060219 PMC9374044

[13] Lambin P Leijenaar RTH Deist TM Peerlings J de Jong EEC van Timmeren J. Radiomics: the bridge between medical imaging and personalized medicine. Nat Rev Clin Oncol. (2017) 14:749–62. doi: 10.1038/nrclinonc.2017.141, PMID: 28975929

[14] Attenberger UI Langs G. How does radiomics actually work?—review. Röfo. (2020) 193:652–7. doi: 10.1055/a-1293-8953, PMID: 33264805

[15] Page MJ McKenzie JE Bossuyt PM Boutron I Hoffmann TC Mulrow CD. The Prisma 2020 statement: An updated guideline for reporting systematic reviews. BMJ. (2021) 372:n71. doi: 10.1136/bmj.n71, PMID: 33782057 PMC8005924

[16] Meng F Yang Y Jin G. Research Progress on Mri for white matter Hyperintensity of presumed vascular origin and cognitive impairment. Front Neurol. (2022) 13:13. doi: 10.3389/fneur.2022.865920, PMID: 35873763 PMC9301233

[17] Karvelas N Elahi FM. White matter Hyperintensities: complex predictor of complex outcomes. J Am Heart Assoc. (2023) 12:e030351. doi: 10.1161/jaha.123.030351, PMID: 37349890 PMC10356075

[18] Alber J Alladi S Bae HJ Barton DA Beckett LA Bell JM. White matter Hyperintensities in vascular contributions to cognitive impairment and dementia (Vcid): knowledge gaps and opportunities. Alzheimers Dement. (2019) 5:107–17. doi: 10.1016/j.trci.2019.02.001, PMID: 31011621 PMC6461571

[19] Herman AL de Havenon A Falcone GJ Prabhakaran S Sheth KN. Racial/ethnic variation in white matter Hyperintensity progression in the accordion mind study. Neurologist. (2022). 28:157–9. doi: 10.1097/nrl.0000000000000454, PMID: 35834785

[20] Seixas AA Turner AD Bubu OM Jean-Louis G de Leon MJ Osorio RS. Obesity and race may explain differential burden of white matter Hyperintensity load. Clin Interv Aging. (2021) 16:1563–71. doi: 10.2147/cia.S316064, PMID: 34465985 PMC8402977

[21] Mok V Srikanth V Xiong Y Phan TG Moran C Chu S. Race-ethnicity and cerebral small vessel disease – comparison between Chinese and white populations. Int J Stroke. (2014) 9:36–42. doi: 10.1111/ijs.12270, PMID: 24661839

[22] Lohner V Pehlivan G Sanroma G Miloschewski A Schirmer MD Stöcker T. Relation between sex, menopause, and white matter Hyperintensities. Neurology. (2022) 99:e935–43. doi: 10.1212/wnl.0000000000200782, PMID: 35768207 PMC9502737

[23] Tamura A Kuriyama N Akazawa K Ozaki E Watanabe I Ohshima Y. A 10-year longitudinal study of deep white matter lesions on magnetic resonance imaging. Neuroradiology. (2021) 63:1599–609. doi: 10.1007/s00234-020-02626-2, PMID: 33599817

[24] Zhang B Wang Y Wang B Chu YH Jiang Y Cui M. Mri-based investigation of association between cerebrovascular structural alteration and white matter Hyperintensity induced by high blood pressure. J Magn Reson Imaging. (2021) 54:1516–26. doi: 10.1002/jmri.27815, PMID: 34184365

[25] Grosu S Lorbeer R Hartmann F Rospleszcz S Bamberg F Schlett CL. White matter Hyperintensity volume in pre-diabetes, diabetes and Normoglycemia. BMJ Open Diabetes Res Care. (2021) 9:2050. doi: 10.1136/bmjdrc-2020-002050, PMID: 34183320 PMC8240582

[26] Park K-I Jung K-H Lee E-J Lee W-J Hwang SA Kim S. Classification of white matter lesions and characteristics of small vessel disease markers. Eur Radiol. (2022) 33:1143–51. doi: 10.1007/s00330-022-09070-1, PMID: 35980432

[27] Busby N Wilson S Wilmskoetter J Newman-Norlund R Sayers S Newman-Norlund S. White matter Hyperintensity load mediates the relationship between age and cognition. Neurobiol Aging. (2023) 132:56–66. doi: 10.1016/j.neurobiolaging.2023.08.007, PMID: 37729770 PMC12573672

[28] Zhang W Cheng Z Fu F Zhan Z. Prevalence and clinical characteristics of white matter hyperintensities in migraine: a meta-analysis. NeuroImage. (2023) 37:312. doi: 10.1016/j.nicl.2023.103312 PMC9827384 36610309

[29] Qi X Pei Y Malone SK Wu B. Social isolation, sleep disturbance, and cognitive functioning (Hrs): a longitudinal mediation study. J Gerontol A Biol Sci Med Sci. (2023) 78:1826–33. doi: 10.1093/gerona/glad004, PMID: 36617184 PMC10562894

[30] Sehrawat P Gupta A Garg A Vishnu V Rajan R Bhatia R. Adult-onset Adrenoleukodystrophy presenting with atypical location of white matter lesions. Neurology. (2022) 99:1051–2. doi: 10.1212/wnl.0000000000201437, PMID: 36180238

[31] Cairns J Vavasour IM Traboulsee A Carruthers R Kolind SH Li DKB. Diffusely abnormal white matter in multiple sclerosis. J Neuroimaging. (2021) 32:5–16. doi: 10.1111/jon.12945, PMID: 34752664

[32] Solé-Guardia G Custers E de Lange A Clijncke E Geenen B Gutierrez J. Association between hypertension and neurovascular inflammation in both normal-appearing white matter and white matter hyperintensities. Acta Neuropathol Commun. (2023) 11:2. doi: 10.1186/s40478-022-01497-3, PMID: 36600303 PMC9811756

[33] Wu Y Ke J Ye S Shan L-L Xu S Guo S-F. 3D visualization of whole brain vessels and quantification of vascular pathology in a chronic hypoperfusion model causing white matter damage. Transl Stroke Res. (2023) 15:659–71. doi: 10.1007/s12975-023-01157-1, PMID: 37222915

[34] Kerkhofs D Wong SM Zhang E Staals J Jansen JFA van Oostenbrugge RJ. Baseline blood-brain barrier leakage and longitudinal microstructural tissue damage in the periphery of white matter Hyperintensities. Neurology. (2021) 96:e2192–200. doi: 10.1212/wnl.0000000000011783, PMID: 33762423 PMC8166427

[35] Lahna D Schwartz DL Woltjer R Black SE Roese N Dodge H. Venous Collagenosis as pathogenesis of white matter Hyperintensity. Ann Neurol. (2022) 92:992–1000. doi: 10.1002/ana.26487, PMID: 36054513 PMC9671829

[36] Gyanwali B Mutsaerts HJMM Tan CS Kaweilh OR Petr J Chen C. Association of Arterial Spin Labeling Parameters with cognitive decline, vascular events, and mortality in a memory-clinic sample. Am J Geriatr Psychiatry. (2022) 30:1298–309. doi: 10.1016/j.jagp.2022.06.007, PMID: 35871110

[37] Li M-J Yeh F-C Huang S-H Huang C-X Zhang H Liu J. Differential Tractography and correlation Tractography findings on patients with mild traumatic brain injury: a pilot study. Front Hum Neurosci. (2022) 16:1902. doi: 10.3389/fnhum.2022.751902, PMID: 35126076 PMC8811572

[38] Xie Y Xie L Kang F Jiang J Yao T Mao G. Association between white matter alterations and domain-specific cognitive impairment in cerebral small vessel disease: a meta-analysis of diffusion tensor imaging. Front Aging Neurosci. (2022) 14:9088. doi: 10.3389/fnagi.2022.1019088, PMID: 36483114 PMC9722766

[39] Bian B Zhou B Shao Z Zhu X Jie Y Li D. Feasibility of diffusion kurtosis imaging in evaluating cervical spinal cord injury in multiple sclerosis. Medicine. (2023) 102:e34205. doi: 10.1097/md.0000000000034205, PMID: 37478237 PMC10662919

[40] Zhu Q Zheng Q Luo D Peng Y Yan Z Wang X. The application of diffusion kurtosis imaging on the heterogeneous white matter in relapsing-remitting multiple sclerosis. Front Neurosci. (2022) 16:16. doi: 10.3389/fnins.2022.849425, PMID: 35360163 PMC8960252

[41] Benitez A Jensen JH Thorn K Dhiman S Fountain-Zaragoza S Rieter WJ. Greater diffusion restriction in white matter in preclinical Alzheimer disease. Ann Neurol. (2022) 91:864–77. doi: 10.1002/ana.26353, PMID: 35285067 PMC9106903

[42] Liu D Ma X Li X Li K Bu Q Zhou L. Correlation study between the microstructural abnormalities of medial prefrontal cortex and white matter Hyperintensities with mild cognitive impairment patients: a diffusion kurtosis imaging study. Psychiatry Res Neuroimaging. (2025) 348:348. doi: 10.1016/j.pscychresns.2025.111958, PMID: 39893732

[43] Andreassen S Lindland EMS Solheim AM Beyer MK Ljøstad U Mygland Å. Cognitive function, fatigue and Fazekas score in patients with acute Neuroborreliosis. Ticks Tick-Borne Dis. (2021) 12:101678. doi: 10.1016/j.ttbdis.2021.101678, PMID: 33529985

[44] Shao Y Chen Z Ming S Ye Q Shu Z Gong C. Predicting the development of Normal-appearing white matter with Radiomics in the aging brain: a longitudinal clinical study. Front Aging Neurosci. (2018) 10:393. doi: 10.3389/fnagi.2018.00393, PMID: 30546304 PMC6279861

[45] Shao Y Ruan J Xu Y Shu Z He X. Comparing the performance of two radiomic models to predict progression and progression speed of white matter hyperintensities. Front Neuroinform. (2021) 15:295. doi: 10.3389/fninf.2021.789295, PMID: 34924990 PMC8671609

[46] Hou J Jin H Zhang Y Xu Y Cui F Qin X. Hybrid model of Ct-fractional flow reserve, Pericoronary fat attenuation index and Radiomics for predicting the progression of Wmh: a dual-center pilot study. Front Cardiovasc Med. (2023) 10:768. doi: 10.3389/fcvm.2023.1282768, PMID: 38179506 PMC10766365

[47] Shu ZY Shao Y Xu YY Ye Q Cui SJ Mao DW. Radiomics nomogram based on MRI for predicting white matter hyperintensity progression in elderly adults. J Magn Reson Imaging. (2019) 51:535–46. doi: 10.1002/jmri.26813, PMID: 31187560

[48] Shu Z Pang P Wu X Cui S Xu Y Zhang M. An integrative nomogram for identifying early-stage Parkinson’s disease using non-motor symptoms and white matter-based radiomics biomarkers from whole-brain MRI. Front Aging Neurosci. (2020) 12:616. doi: 10.3389/fnagi.2020.548616, PMID: 33390927 PMC7773758

[49] Ortiz-Ramón R Valdés Hernández MC González-Castro V Makin S Armitage PA Aribisala BS. Identification of the presence of ischaemic stroke lesions by means of texture analysis on brain magnetic resonance images. Comput Med Imaging Graph. (2019) 74:12–24. doi: 10.1016/j.compmedimag.2019.02.006, PMID: 30921550 PMC6553681

[50] Gupta R Bilgin C Jabal MS Kandemirli S Ghozy S Kobeissi H. Quality assessment of Radiomics studies on functional outcomes after acute ischemic stroke–a systematic review. World Neurosurg. (2024) 183:164–71. doi: 10.1016/j.wneu.2023.11.154, PMID: 38056625

[51] Serrano E Moreno J Llull L Rodríguez A Zwanzger C Amaro S. Radiomic-based nonlinear supervised learning classifiers on non-contrast Ct to predict functional prognosis in patients with spontaneous intracerebral hematoma. Radiología. (2023) 65:519–30. doi: 10.1016/j.rxeng.2023.08.002, PMID: 38049251

[52] Feigin VL Stark BA Johnson CO Roth GA Bisignano C Abady GG. Global, regional, and National Burden of stroke and its risk factors, 1990–2019: a systematic analysis for the global burden of disease study 2019. Lancet Neurol. (2021) 20:795–820. doi: 10.1016/s1474-4422(21)00252-0, PMID: 34487721 PMC8443449

[53] Giese AK Schirmer MD Dalca AV Sridharan R Donahue KL Nardin M. White matter Hyperintensity burden in acute stroke patients differs by ischemic stroke subtype. Neurology. (2020) 95:e79–88. Epub 2020/06/05. doi: 10.1212/wnl.0000000000009728, PMID: 32493718 PMC7371377

[54] Guo Y Yang Y Cao F Li W Wang M Luo Y. Novel survival features generated by clinical text information and Radiomics features may improve the prediction of ischemic stroke outcome. Diagnostics. (2022) 12:664. doi: 10.3390/diagnostics12071664, PMID: 35885568 PMC9324145

[55] Tang T-y Jiao Y Cui Y Zhao D-l Zhang Y Wang Z. Penumbra-based radiomics signature as prognostic biomarkers for thrombolysis of acute ischemic stroke patients: a multicenter cohort study. J Neurol. (2020) 267:1454–63. doi: 10.1007/s00415-020-09713-7, PMID: 32008072

[56] Guo K Zhu B Li R Xi J Wang Q Chen K. Machine learning-based nomogram: integrating Mri Radiomics and clinical indicators for prognostic assessment in acute ischemic stroke. Front Neurol. (2024) 15:9031. doi: 10.3389/fneur.2024.1379031, PMID: 38933326 PMC11202100

[57] Xia Y Li L Liu P Zhai T Shi Y. Machine learning prediction model for functional prognosis of acute ischemic stroke based on Mri Radiomics of white matter Hyperintensities. BMC Med Imaging. (2025) 25:91. doi: 10.1186/s12880-025-01632-1, PMID: 40108506 PMC11924691

[58] Bonkhoff AK Hong S Bretzner M Schirmer MD Regenhardt RW Arsava EM. Association of Stroke Lesion Pattern and White Matter Hyperintensity Burden with stroke severity and outcome. Neurology. (2022) 99:e1364–79. doi: 10.1212/wnl.0000000000200926, PMID: 35803717 PMC9576289

[59] Bretzner M Bonkhoff AK Schirmer MD Hong S Dalca AV Donahue KL. Mri Radiomic signature of white matter Hyperintensities is associated with clinical phenotypes. Front Neurosci. (2021) 15:244. doi: 10.3389/fnins.2021.691244, PMID: 34321995 PMC8312571

[60] Pasi M Cordonnier C. Clinical relevance of cerebral small vessel diseases. Stroke. (2020) 51:47–53. doi: 10.1161/strokeaha.119.024148, PMID: 31752613

[61] Grau-Olivares M Arboix A. Mild cognitive impairment in stroke patients with ischemic cerebral small-vessel disease: a forerunner of vascular dementia? Expert Rev Neurother. (2009) 9:1201–17. Epub 2009/08/14. doi: 10.1586/ern.09.73, PMID: 19673608

[62] Rudilosso S Rodríguez-Vázquez A Urra X Arboix A. The potential impact of neuroimaging and translational research on the clinical management of lacunar stroke. Int J Mol Sci. (2022) 23:497. doi: 10.3390/ijms23031497, PMID: 35163423 PMC8835925

[63] Scheltens P De Strooper B Kivipelto M Holstege H Chételat G Teunissen CE. Alzheimer’s disease. Lancet. (2021) 397:1577–90. doi: 10.1016/s0140-6736(20)32205-4, PMID: 33667416 PMC8354300

[64] Leandrou S Lamnisos D Bougias H Stogiannos N Georgiadou E Achilleos KG. A cross-sectional study of explainable machine learning in Alzheimer’s disease: diagnostic classification using MR radiomic features. Front Aging Neurosci. (2023) 15:871. doi: 10.3389/fnagi.2023.1149871, PMID: 37358951 PMC10285704

[65] Tang L Wu X Liu H Wu F Song R Zhang W. Individualized prediction of early Alzheimer’s disease based on magnetic resonance imaging Radiomics, clinical, and laboratory examinations: a 60-month follow-up study. J Magn Reson Imaging. (2021) 54:1647–57. doi: 10.1002/jmri.27689, PMID: 33987915

[66] Dadar M Mahmoud S Zhernovaia M Camicioli R Maranzano J Duchesne S. White matter hyperintensity distribution differences in aging and neurodegenerative disease cohorts. NeuroImage. (2022) 36:3204. doi: 10.1016/j.nicl.2022.103204 PMC9668605 36155321

[67] Garnier-Crussard A Bougacha S Wirth M Dautricourt S Sherif S Landeau B. White matter hyperintensity topography in Alzheimer’s disease and links to cognition. Alzheimers Dement. (2021) 18:422–33. doi: 10.1002/alz.12410, PMID: 34322985 PMC9292254

[68] Fiford CM Sudre CH Pemberton H Walsh P Manning E Malone IB. Automated white matter hyperintensity segmentation using Bayesian model selection: assessment and correlations with cognitive change. Neuroinformatics. (2020) 18:429–49. doi: 10.1007/s12021-019-09439-6, PMID: 32062817 PMC7338814

[69] Chen K Jin Z Fang J Qi L Liu C Wang R. The impact of cerebral small vessel disease burden and its imaging markers on gait, postural control, and cognition in Parkinson’s disease. Neurol Sci. (2022) 44:1223–33. doi: 10.1007/s10072-022-06563-1, PMID: 36547777

[70] Hou M Hou X Qiu Y Wang J Zhang M Mao X. Characteristics of cognitive impairment and their relationship with total cerebral small vascular disease score in Parkinson’s disease. Front Aging Neurosci. (2022) 14:506. doi: 10.3389/fnagi.2022.884506 PMC9301002 35875803

[71] Kang JJ Chen Y Xu GD Bao SL Wang J Ge M. Combining quantitative susceptibility mapping to Radiomics in diagnosing Parkinson’s disease and assessing cognitive impairment. Eur Radiol. (2022) 32:6992–7003. doi: 10.1007/s00330-022-08790-8, PMID: 35461376

[72] Ben Bashat D Thaler A Lerman Shacham H Even-Sapir E Hutchison M Evans KC. Neuromelanin and T2*-Mri for the assessment of genetically at-risk, prodromal, and symptomatic Parkinson’s disease. Npj. Parkinsons Dis. (2022) 8:139. doi: 10.1038/s41531-022-00405-9, PMID: 36271084 PMC9586960

[73] Li J Liu X Wang X Liu H Lin Z Xiong N. Diffusion tensor imaging Radiomics for diagnosis of Parkinson’s disease. Brain Sci. (2022) 12:851. doi: 10.3390/brainsci12070851, PMID: 35884658 PMC9313106

[74] Nada A. Advances in Parkinson’s disease diagnosis through diffusion kurtosis imaging and Radiomics. Acad Radiol. (2025) 32:930–1. doi: 10.1016/j.acra.2024.12.048, PMID: 39741053

[75] Sun J Cong C Li X Zhou W Xia R Liu H. Identification of Parkinson’s disease and multiple system atrophy using multimodal PET/MRI radiomics. Eur Radiol. (2023) 34:662–72. doi: 10.1007/s00330-023-10003-9, PMID: 37535155

[76] Huang X Wen MC Ng SYE Hartono S Chia NSY Choi X. Periventricular white matter Hyperintensity burden and cognitive impairment in early Parkinson’s disease. Eur J Neurol. (2020) 27:959–66. doi: 10.1111/ene.14192, PMID: 32124496

[77] Wu H Hong H Wu C Qin J Zhou C Tan S. Regional white matter Hyperintensity volume in Parkinson’s disease and associations with the motor signs. Ann Clin Transl Neurol. (2023) 10:1502–12. doi: 10.1002/acn3.51839, PMID: 37353980 PMC10502622

[78] Liu H Deng B Xie F Yang X Xie Z Chen Y. The influence of white matter Hyperintensity on cognitive impairment in Parkinson’s disease. Ann Clin Transl Neurol. (2021) 8:1917–34. doi: 10.1002/acn3.51429, PMID: 34310081 PMC8419402

[79] Rektor I Svátková A Vojtíšek L Zikmundová I Vaníček J Király A. White matter alterations in Parkinson’s disease with Normal cognition precede Grey matter atrophy. PLoS One. (2018) 13:e0187939. doi: 10.1371/journal.pone.0187939, PMID: 29304183 PMC5755732

[80] Shu ZY Cui SJ Wu X Xu Y Huang P Pang PP. Predicting the progression of Parkinson’s disease using conventional MRI and machine learning: an application of radiomic biomarkers in whole-brain white matter. Magn Reson Med. (2020) 85:1611–24. doi: 10.1002/mrm.28522, PMID: 33017475

[81] Haliasos N Giakoumettis D Gnanaratnasingham P Low HL Misbahuddin A Zikos P. Personalizing deep brain stimulation therapy for Parkinson’s disease with whole-brain MRI radiomics and machine learning. Cureus. (2024) 16. doi: 10.7759/cureus.c195 PMC11161197 38854362

[82] Tubi MA Feingold FW Kothapalli D Hare ET King KS Thompson PM. White matter Hyperintensities and their relationship to cognition: effects of segmentation algorithm. NeuroImage. (2020) 206:327. doi: 10.1016/j.neuroimage.2019.116327, PMID: 31682983 PMC6981030

